# Amplification of overlapping DNA amplicons in a single-tube multiplex PCR for targeted next-generation sequencing of *BRCA1* and *BRCA2*

**DOI:** 10.1371/journal.pone.0181062

**Published:** 2017-07-12

**Authors:** Desiree Schenk, Gang Song, Yue Ke, Zhaohui Wang

**Affiliations:** Department of Research and Development, Pillar Biosciences, Natick, Massachusetts, United States of America; ICELAND

## Abstract

Current PCR-based target enrichment methods for next generation sequencing (NGS) of overlapping amplicons often requires separate PCR reactions and subsequent pooling of amplicons from the different reactions. The study presents a novel method, deemed stem-loop inhibition mediated amplification (SLIMamp), for amplifying overlapping or tiled amplicons in a single multiplex PCR reaction. During a SLIMamp PCR reaction, a stem loop structure formed by the overlapping amplicon suppresses additional amplification of itself by preventing the annealing of the primers. Using the SLIMamp strategy, we designed a next-generation sequencing (NGS) assay to enrich the exon regions of *BRCA1* and *BRCA2* for sequencing on an Illumina MiSeq system. We used 35 cell line DNAs and 6 patient blood DNAs in the study to evaluate the assay performance. For each sample, all targeted regions were successfully amplified and sequenced with excellent coverage uniformity and specificity. >99% of the total sequencing reads were mapped to the human reference genome (hg19) and >99% of the mapped reads were on the targeted exons. >98% of bases were covered at >0.20x of the mean coverage and >99% are covered at >0.15x of the mean depth. Among 34 independently sequenced samples, all variants were reliably detected with no false positives or false negatives. SLIMamp provides a robust method for single-tube multiplex PCR amplification of numerous, overlapping amplicons that tile for targeted next-generation sequencing.

## Introduction

Target enrichment methods in next generation sequencing can be categorized into two main classes: hybrid capture enrichment and amplicon-based enrichment[1–3]. Hybrid capture based methods such as SureSelect (Agilent Technologies) and SeqCap (Roche Nimblegen) are highly scalable and have advantages for large gene panels and whole exome sequencing[4,5]. However, hybrid capture methods typically require high DNA input amounts, a complicated and lengthy library preparation process, and high cost.

Amplicon-based target enrichment strategies can be broadly classified into the following three categories: hybridization-extension-ligation amplicon enrichment, anchored multiplex PCR (AMP), and PCR-based enrichment. Hybridization-extension-ligation amplicon enrichment methods include Haloplex (Agilent) and TruSeq Amplicon (Illumina). One long, looped oligo (HaloPlex) or two-tagged oligos (TruSeq) are hybridized to the flanking sequences of the targeted region of interest followed by extension and ligation to fill the gaps between the hybridization sites. The resulting products are then indexed and amplified by PCR using common primers. These methods require a relatively high DNA input amount and significant hands-on manipulation [6,7]. In AMP (ArcherDx), the multiplex PCR uses one primer specific to the target—the “anchor”—and another common primer that binds to the universal adaptor that has been ligated to the fragmented template. This approach is most effective for detecting gene rearrangements without prior knowledge of the fusion partners using cDNA as input. However, it often needs an additional anchored primer for each target for semi-nested PCR to increase the PCR specificity [8].

**Fig 1:**
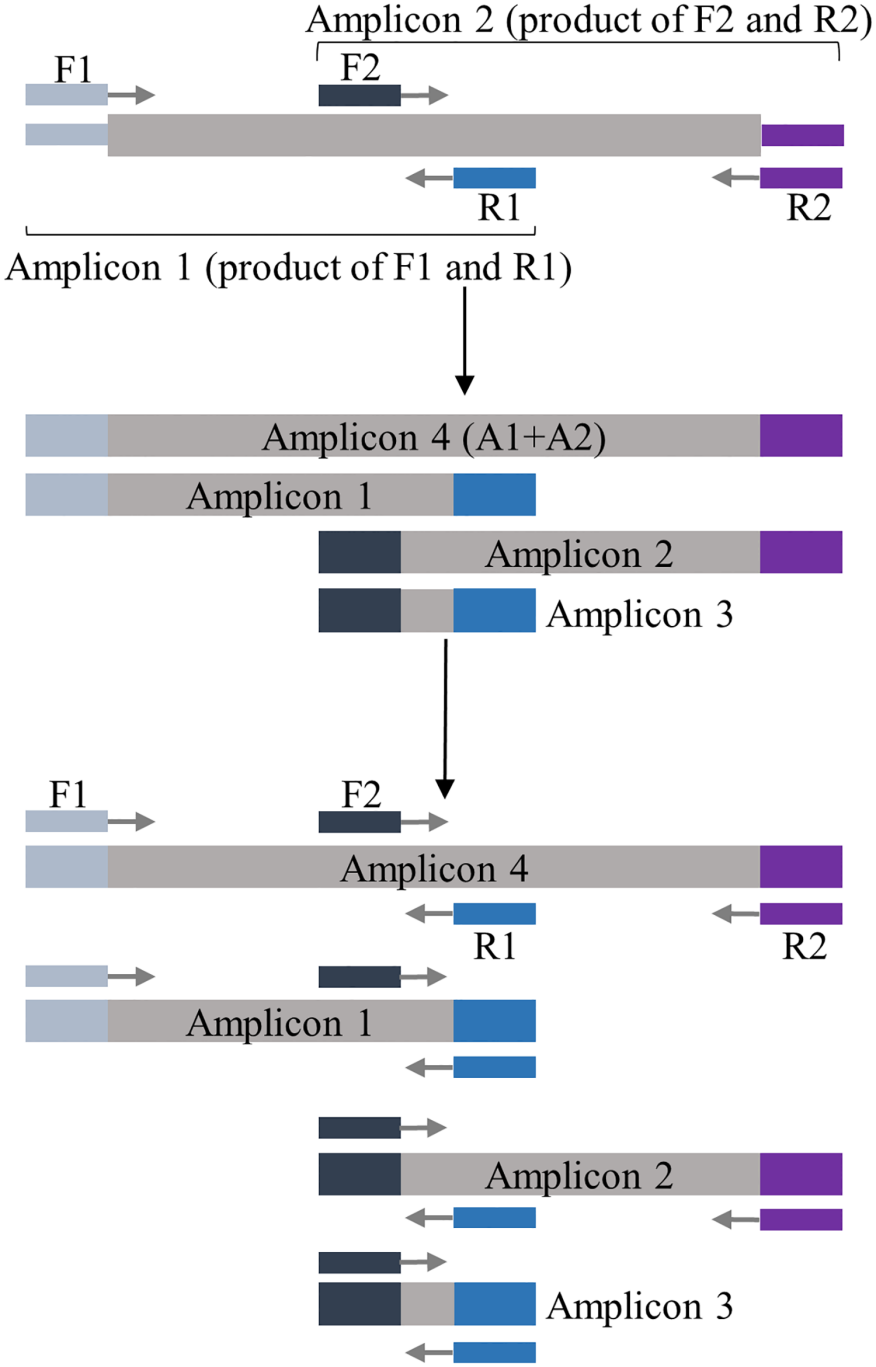
Conventional multiplex PCR. During conventional multiplex PCR with overlapping amplicons, four amplicons are produced. For each cycle, Amplicon 3 can be amplified from not only the original template, but also Amplicons 1, 2, and 4. Its growth overtakes the reaction and hinders the amplification of the target amplicons.

Target enrichment by PCR such as AmpliSeq (Thermo Fisher Scientific), GeneRead (Qiagen) and Multiplicom can generate deep sequencing coverage using very little DNA with straight-forward and faster processes. This approach is highly efficient in targeting the hotspots of somatic mutations. However, PCR enrichment of long target regions such as the entire coding sequences of genes require multiple reactions to separately amplify the overlapping amplicons that tile the entire target sequences. When all primers are present in one reaction, the overlapping regions between the adjacent overlapping amplicons will be preferentially amplified and dominate the reaction, resulting in the drop-out of the actual targeted amplicons and gaps in sequencing coverage (Fig 1).

To overcome the limitation described above, we have developed a one-tube gene-specific multiplex PCR (mPCR) based method, SLIMamp (Stem-Loop Inhibition Mediated Amplification), that enables the enrichment of target amplicons over the overlapping regions of amplicons. We applied this strategy to amplify the coding sequences of both *BRCA1* and *BRCA2* in a single multiplex reaction for targeted sequencing. The *BRCA1* and *BRCA2* SLIMamp assay utilizes 91 amplicons, 53 of which overlap with at least one adjacent amplicon. The prepared libraries were sequenced on Illumina’s MiSeq instrument, and the SLIMamp assay performance was assessed with previously validated samples.

## Materials and methods

### Primer design

#### 2-plex primer design

**Fig 2:**
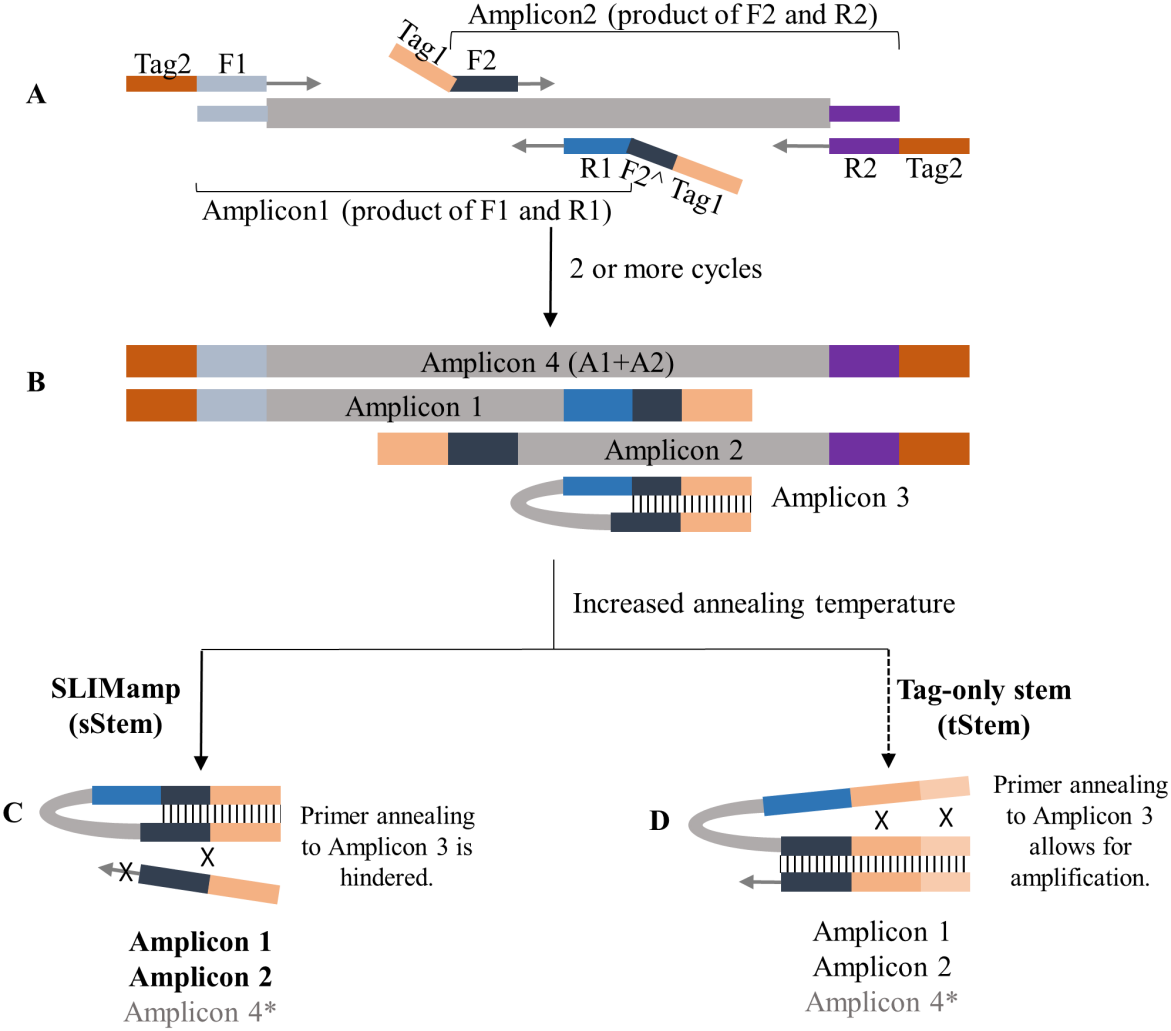
SLIMamp vs Tag-only stem. A) During the initial cycling, the annealing temperature used is for gene-specific primer annealing and extension. B) After at least two cycles, the amplicons are fully formed with tags on both ends. Amplicon 3 forms a stem-loop structure. C) PCR products with SLIMamp reactions (Amplicons 1, 2, and 4). SLIMamp inhibits the amplification of Amplicon 3 by forming a stem that hinders the hybridization of the primers. When the stem forms in SLIMamp, only a fraction of F2 is available for primer annealing. The major amplification products are the intended targets (Amplicons 1 and 2) rather than the overlap (Amplicon 3) with little of Amplicon 4. D) PCR products (Amplicons 1, 2, 3, and 4) with tag-only stem reactions. Even with the formation of a stem by increasing the tag length, the full sequence of F2 is contained in the loop structure, allowing the annealing of the primer to outcompete the hybridization of the stem. Amplicons 1, 2, and 3 are amplified with very little of Amplicon 4. *The efficiency of Amplicon 4 is less than that of Amplicons 1 and 2 due to its longer length.

**Fig 3:**
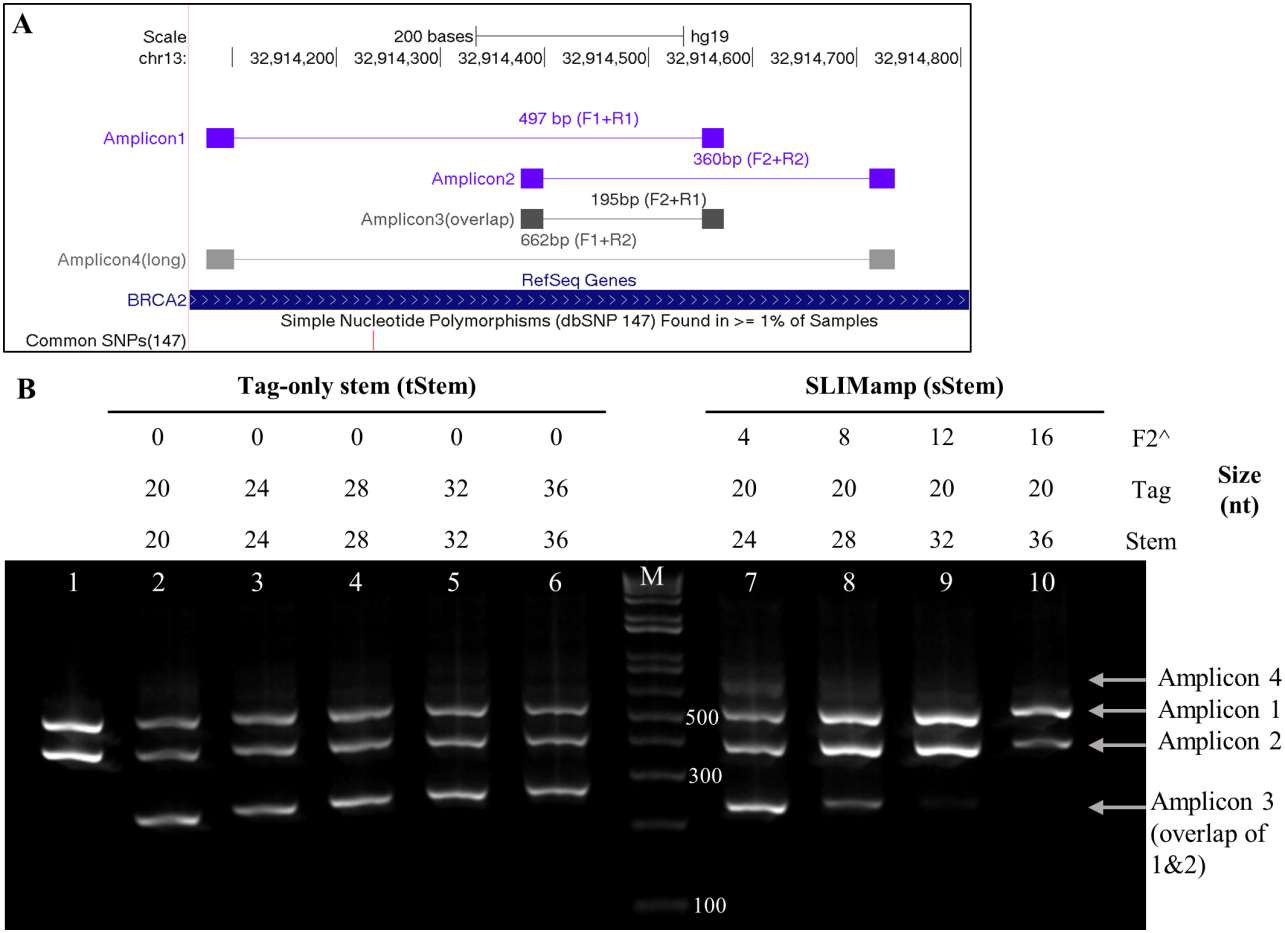
Tag-only stem and SLIMamp 2-plex PCR. A) Four amplicons are produced during the amplification of two overlapping amplicons. B) Gel electrophoresis of two oligo sets (tStem and sStem) that form stem-loop structures. Lane M: 1Kb Plus ladder. Lane 1 is a singleplex control where Amplicon 1 and 2 were amplified separately and combined for electrophoresis. Lanes 2–6: mPCR with tag-only stem (tStem). The F2 and R1 primers form a stem, but the entire length of F2 for the overlapping amplicon is available for annealing in the loop of the stem-loop structure. Lanes 7–10: mPCR with SLIMamp primers. The stem length is similar to lanes 3–6, but the stem is formed with F2, creating competition for annealing with the primers. Amplicon 3 was greatly inhibited in the SLIMamp reactions but not in any of the tStem reactions.

For the initial proof-of-concept 2-plex SLIMamp study (design overview of SLIMamp in Fig 2A–2C), the gene-specific PCR primers of two amplicons, Amplicon 1 (A1, 497 bp) and Amplicon 2 (A2, 360 bp) with a 195 bp overlapping region (A3, Fig 3A), were designed using Primer 3 (http://bioinfo.ut.ee/primer3-0.4.0/primer3/) at default settings with primer melting temperature (Tm) at 60°C ± 3°C. The 5’-end of the forward and reverse primers were tagged with either caacgatcgtcgaaattcgc (t1) or tacacgacgctcttccgatct (t2). The t1 and t2 are the tag sequences used in Illumina’s TrueSeq Custom Amplicon (TSCA) oligoes and serve as the primer binding sites of the universal indexing PCR primers in TSCA library preparation. For each targeted amplicon (A1 and A2), the forward and reverse primers were tagged with different tag sequences.

In the SLIMamp design, the forward primer of Amplicon 2 (F2) and reverse primer of Amplicon 1 (R1), which amplify the short overlapping amplicon (Amplicon 3), were tagged at the 5’-ends with the same tag sequences (t1). Then various lengths (4, 8, 12, and 16 nucleotides (nt)) of the 5’-end part of the F2 (F2^) were inserted between the gene-specific R1 sequence and its 5’-end tag sequence (t1) to study the SLIMamp efficiency (Table 1).

**Table 1:** 2-Plex primers.

	Oligo ID	Sequence*	Amplicon
**Gene-specific primers with tags**	**t2_F1**	tacacgacgctcttccgatct GTGAAAGACATATTTACAGACAGTTTC	Amplicon1
	**t1_R1**	caacgatcgtcgaaattcgc CTTGTGAGCTGGTCTGAATGT	Amplicon1
	**t1_F2**	caacgatcgtcgaaattcgc AGGGAAGCTTCATAAGTCAGTC	Amplicon2
	**t2_R2**	tacacgacgctcttccgatct TCCTCTAACACTCCCTTAACTTTGT	Amplicon2
**SLIMamp**	**t1_F2^4_R1**	caacgatcgtcgaaattcgc AGGG CTTGTGAGCTGGTCTGAATGT	Amplicon1
	**t1_F2^8_R1**	caacgatcgtcgaaattcgc AGGGAAGC CTTGTGAGCTGGTCTGAATGT	Amplicon1
	**t1_F2^12_R1**	caacgatcgtcgaaattcgc AGGGAAGCTTCA CTTGTGAGCTGGTCTGAATGT	Amplicon1
	**t1_F2^16_R1**	caacgatcgtcgaaattcgc AGGGAAGCTTCATAAG CTTGTGAGCTGGTCTGAATGT	Amplicon1
**Tag-only stem (tStem)**	**4nt_t1_R1**	AGGG caacgatcgtcgaaattcgc CTTGTGAGCTGGTCTGAATGT	Amplicon1
	**8nt_t1_R1**	AGGGAAGC caacgatcgtcgaaattcgc CTTGTGAGCTGGTCTGAATGT	Amplicon1
	**12nt_t1_R1**	AGGGAAGCTTCA caacgatcgtcgaaattcgc CTTGTGAGCTGGTCTGAATGT	Amplicon1
	**16nt_t1_R1**	AGGGAAGCTTCATAAG caacgatcgtcgaaattcgc CTTGTGAGCTGGTCTGAATGT	Amplicon1
	**4nt_t1_F2**	AGGG caacgatcgtcgaaattcgc AGGGAAGCTTCATAAGTCAGTC	Amplicon2
	**8nt_t1_F2**	AGGAAGC caacgatcgtcgaaattcgc AGGGAAGCTTCATAAGTCAGTC	Amplicon2
	**12nt_t1_F2**	AGGGAAGCTTCA caacgatcgtcgaaattcgc AGGGAAGCTTCATAAGTCAGTC	Amplicon2
	**16nt_t1_F2**	AGGGAAGCTTCATAAG caacgatcgtcgaaattcgc AGGGAAGCTTCATAAGTCAGTC	Amplicon2

In the 2-plex study, both tag-only stem primers and SLIMamp primers were used to study the inhibition of the amplification of the overlapping amplicon.

*Lower case indicates tag sequences; Underline indicates inserted partial Forward primer sequences from the next amplicon; un-labeled, upper case sequences are gene-specific sequences.

To demonstrate that distinct property and effectiveness of the SLIMamp design from simple, direct stem-loop forming primers, we maintained the same t1 tag sequences for F1 and R2 as in SLIMamp and then simply extended the tag sequences at their 5’-ends by various lengths to increase the stem size. For direct comparison, the 5’-end extended tag sequences and lengths matched the F2^ used in the SLIMamp design (Table 1).

#### BRCA1 and BRCA2 SLIMamp primer design

To cover the region of interest (ROI), 91 amplicons were designed using the SLIMamp approach (S1 Table). The ROI includes the coding regions ± 20 bp (17,769 bp total) for both *BRCA1* and *BRCA2* (NM_007294.3 and NM_000059.3, respectively). The primers were designed such that no SNPs with a minor allele frequency above 0.09% are present in the last ten nucleotides of the 3’-ends of the oligonucleotides. Out of the 91 amplicons, 53 of the amplicons overlap with at least one adjacent amplicon, resulting in 45 overlapped regions that range from 49 bp to 193 bp in size (S1 Table). F2^ or R1^ lengths in the primers covering overlapping amplicons varied from 7–14 nucleotides (see explanation of F2^ and R1^ in Design Overview in Results). All primers were synthesized by IDT (Ames, IA).

### 2-plex PCR of overlapping amplicons

Reactions were prepared using 10 ng of DNA (Promega, Madison, WI), 2x Kapa2G Fast ReadyMix (Kapa Biosystems, Wilmington, MA) and 0.2 μM each of the appropriate PCR primers (IDT; Ames, IA, Table 2 and Fig 3B). Cycling was performed as follows: one cycle of 95°C for 2 minutes, 5 cycles of 95°C for 30 s and 60°C for 90 s, and 30 cycles of 95°C for 30 s and 72°C for 90 s. The products were visualized by performing electrophoresis with a 2% Agarose E-Gel EX (Thermo Fisher Scientific, Waltham, MA). The E-Gel 1Kb Plus DNA Ladder was used as the size marker (Thermo Fisher Scientific, Waltham, MA).

**Table 2:** 2-Plex PCR primers used in Fig 3B.

Lane ID	Name	Multiplex Primer Mix				F2_R1 Short Amplicon
		Amplicon1		Amplicon2		Loop Length(nt); Stem Oligo; Stem length(nt)
**1**	SinglePlex Control mix^a^					no stem loop
**2**	tStem_t1	t2_F1	t1_R1	t1_F2	t2_R2	195; t1_only; 20
**3**	tStem_4nt-t1	t2_F1	4nt-t1_R1	4nt-t1_F2	t2_R2	195; t1+4nt; 24
**4**	tStem_8nt-t1	t2_F1	8nt-t1_R1	8nt-t1_F2	t2_R2	195; t1+8nt; 28
**5**	tStem_12nt-t1	t2_F1	12nt-t1_R1	12nt-t1_F2	t2_R2	195; t1+12nt; 32
**6**	tStem_16nt-t1	t2_F1	16nt-t1_R1	16nt-t1_F2	t2_R2	195; t1+16nt; 36
**Marker**	1Kb Plus Ladder					Not applicable
**7**	sStem_t1-F2^4	t2_F1	t1-F2^4_R1	t1_F2	t2_R2	195; t1+4nt of F2; 24
**8**	sStem_t1-F2^8	t2_F1	t1-F2^8_R1	t1_F2	t2_R2	195; t1+8nt of F2; 28
**9**	sStem_t1-F2^12	t2_F1	t1-F2^12_R1	t1_F2	t2_R2	195; t1+12nt of F2; 32
**10**	sStem_t1-F2^16	t2_F1	t1-F2^16_R1	t1_F2	t2_R2	195; t1+16nt of F2; 36

The reactions in the gel electrophoresis in Fig 3B contains tStem primers from Table 1 in Lanes 2–6 and sStem primers in lanes 7–10.

^a^Contains each singleplex product from separate amplifications mixed at an equal volume ratio.

### Library preparation of BRCA1 and BRCA2 using SLIMamp

#### Gene-specific PCR

To evaluate the SLIMamp assay performance, including coverage statistics, reproducibility, and run capacity, we used 41 samples: 35 DNA samples were obtained from Coriell Biorepository (Camden, NJ) and 6 were anonymized clinical samples. The archived samples were collected using patient informed consent, and the clinical samples were de-identified remnant samples.

To determine the accuracy of variant detection, we assessed the degree of concordance of the variants detected by SLIMamp in NGS with the results obtained with Sanger sequencing and TruSeq Custom Amplicon (TSCA) (Illumina, San Francisco, CA) in 33 of the samples and Genome in a Bottle (GIAB, NA12878). Seven of the samples used for assay performance were not include in the variant concordance study. They consist of 3 Coriell DNA samples that have been reported to be BRCA negative for pathogenic variants (sample details in S2 Table), and four of the clinical samples with limited quantity were not sequenced by Sanger.

For a DNA input study, 28 libraries were prepared using four Coriell DNA samples using seven different input amounts: 5, 10, 20, 30, 50, 75, and 100 ng of DNA. The libraries for the other studies used inputs of 10 or 30 ng of DNA. Gene-specific multiplex PCR was performed using one pool of SLIMamp primers and Pillar Biosciences’ Multiplex PCR MasterMix. The initial 5 cycles were performed with an annealing/extension step at 60°C for gene-specific primer binding/extension. The subsequent PCR cycles were performed with the annealing/extension at 72°C.

#### Universal indexing PCR and NGS

Each SLIMamp product was purified with Agencourt AMPure XP beads (Beckman Coulter, Danvers, MA) and eluted with 32 μL ddH_2_O. The purified products were mixed with Illumina’s TSCA Indexing primers (Illumina, San Francisco, CA) and Pillar Biosciences’ Indexing MasterMix and cycled per Illumina’s instructions using 5 or 7 cycles. The indexed libraries were subsequently purified with AMPure XP beads, quantified using the Qubit High Sensitivity dsDNA Assay (Thermo Fisher Scientific, Waltham, MA), normalized, and pooled. Following the manufacturer’s instructions, the pooled libraries were sequenced on an Illumina MiSeq (MiSeq v2 kit or MiSeq Nano v2 kit) using a 2 x 250 bp paired-end sequencing protocol. The products were visualized by performing electrophoresis with a 2% Agarose SizeSelect E-Gel (Thermo Fisher Scientific, Waltham, MA). The E-Gel 1Kb Plus DNA Ladder was used as the size marker (Thermo Fisher Scientific, Waltham, MA).

### NGS data analysis

The raw sequencing data were demultiplexed and converted to Fastq files by MiSeq Reporter Version 2.5.1. The reads were then aligned to human genome assembly 37/hg19 reference sequence by BWA-MEM [9]. To capture indels missed by BWA-MEM, the un-aligned ends of the mapped reads were locally re-aligned by the Smith–Waterman algorithm using Biopython (https://github.com/biopython/biopython). The paired-end reads were merged based on mapped positions into consensus sequences weighted by phred qualities. The primer regions were then soft-clipped from the merged reads at both ends. The quality-weighted frequency noise for each segment was calculated from all reads covering the segment. Then, the variants on the base positions with quality-weighted frequency of 6 standard deviations above the noise were identified as potential positives. The variants with less than 20 reads supporting the call and less than 20% frequency were filtered out. The remaining variants were annotated using Variant Effect Predictor (version 84, http://grch37.ensembl.org/Homo_sapiens/Tools/VEP) based on the HGVS standard. The segment coverage statistics were calculated from the merged reads. The mapping and on-target rates were obtained using Samtools (https://github.com/samtools/samtools).

## Results

### Design overview

Gene-specific PCR primers with or without 5’-tag sequences designed by conventional methods do not allow the multiplexing of overlapping amplicons in one reaction as illustrated in Fig 1. Fig 1 uses the example of amplifying two targeted overlapping amplicons. F1 and R1 are the gene-specific primers for Amplicon 1, and F2 and R2 are the primers for Amplicon 2. Combining the four PCR primers in one mPCR reaction produces four products: Amplicon 1 by the F1/R1 pair, Amplicon 2 by the F2/R2 pair, a long amplicon spanning the entire region (Amplicon 4) by the F1/R2 pair, and a small amplicon containing the overlapped regions between Amplicons 1 and 2 (Amplicon 3) by the F2/R1 pair (Fig 1). Using PCR conditions with a constant annealing temperature during cycling, the longest amplicon (Amplicon 4) serves as DNA template for all four amplicons' amplification, and each of the two targeted amplicons (Amplicons 1 and 2) can serve as a DNA template for amplification for its self and the overlapping amplicon (Amplicon 3). Consequently, at PCR cycle n, the amplification fold of each of the four products—Amplicon 1, Amplicon 2, Amplicon 4, and Amplicon 3- will be n x 2^n^, n x 2^n^, 2^n^, and n^2^ x 2^n^, respectively, assuming all amplifications occur at 100% efficiency. The amount of the overlapping amplicon (Amplicon 3) is n times higher than that of each of the two targeted amplicons (Amplicon 1 and Amplicon 2), which in turn is n times higher than the amount of the longest amplicon (Amplicon 4). In addition, short amplicons tend to be amplified more efficiently, potentially inhibiting the amplification of the desired DNA sequences by the massive consumption of primers and other reagents.

In the SLIMamp design (Fig 2A), to prevent the amplification of the short, overlapping amplicon (Amplicon 3) by the primer pair F2/R1, the same Tag1 sequences are attached at the 5’-ends of both primers; in addition, a portion of the 5’-end sequence of F2, denoted F2^, is introduced directly between the gene-specific sequence of R1 and its tag (tag1) sequence. Equivalently, a portion of R1 (named R1^) can be inserted between the gene-specific sequence of F2 and its tag sequence. For ease of explanation, we show it using the F2^ orientation only (Fig 2A). At first, a low number of PCR cycles is performed at an annealing temperature allowing the binding of gene-specific primers with the templates. All four amplicons, Amplicons 1, 2, 3 (overlap), and 4 (long), are produced in full-length with corresponding tag sequences at both ends of each amplicon (Fig 2B). Particularly, in the overlapping Amplicon (Amplicon 3), one end contains Tag1 and F2 sequences and the other end contains the complementary sequences of F2^ and Tag1. Consequently, at the subsequent annealing steps during cycling (Fig 2C), the Tag1-F2^ complementary pair sequences in Amplicon 3 form a strong stem, designated as sStem (SLIMamp stem), that renders the sequences inaccessible for the hybridization of the forward primer (Tag1-F2), thus preventing Amplicon 3 from serving as a template for further amplification. The key factor in sStem is the appropriate length of F2^ sequences. When F2^ is not present (F2^ = 0) with a tag-only stem (tStem), increasing tStem stability by extending the tag sequences is ineffective in preventing the primer (Tag1-F2) binding and further amplification occurs (Fig 2D), largely due to the whole F2 gene-specific sequence in Amplicon 3 being in the loop structure that is accessible for further primer annealing. In addition, the annealing temperature of the subsequent cycling is increased to prevent the binding of F2/R1 (Amplicon 3’s primer pair) to the gene-specific sequences in the other three longer amplicons. Together, the amplification of the short overlapping amplicon (Amplicon 3) is greatly inhibited in SLIMamp. Moreover, longer amplicons (Amplicon 4) are typically amplified at lower PCR efficiencies; therefore, the main products in SLIMamp will be the targeted Amplicons 1 and 2.

### Amplification using two overlapping amplicons as a mPCR model

To study the mechanism and the efficiency of SLIMamp PCR, we designed gene-specific primers (Tm ≈ 60°C) for two overlapping amplicons on a region of the *BRCA2* gene, Amplicons 1 (F1+ R1, 497 bp) and 2 (F2 +R2, 360 bp) with a 195 bp overlapping region (Fig 3A). In the SLIMamp design, different lengths of F2^ (F2 = 22 nt), ranging from 4 to 16 nt, were inserted into the R1 primer to form the sStem with the sequence of Tag1-F2^ (24 to 36 nt long) in the overlapping amplicon (Amplicon 3). In the tStem (tag-only stem) design, the F2 and R1 primers were both tailed with extended tag sequences to form the tStem without F2^. For direct comparison, the 5’-end extended tag sequences and lengths in the tStem matched the F2^ in SLIMamp (Tables 1 and 2 and Fig 3B). Multiplex PCR was performed using 2-stage cycling conditions with SLIMamp primers or tStem primers. The PCR products were visualized on a 2% agarose gel (Fig 3B). The mPCR reactions (Lanes 2–10) produced low or undetectable quantities of the longest amplicon (Amplicon 4, ~700 bp with tags), indicating very low PCR efficiency for the long amplicon. Amplicon 3 was detected abundantly and equally in all the tStem PCR reactions in Lanes 2–6 (Fig 3B) regardless of the increased stem length. On the other hand, the Amplicon 3 product gradually decreased while Amplicons 1 and 2 increased in the SLIMamp reactions with the increasing length of F2^ (Lanes 7–10, Fig 3B). Nearly complete Amplicon 3 inhibition was achieved when F2^ ≥12 nt (Lanes 9 and 10, Fig 3B). The results suggested that the key factor to prevent the amplification of the overlapping amplicons in the design was rather the inclusion of F2^ than simply the high stability of the stem.

### SLIMamp *BRCA1* and *BRCA2* library preparation and sequencing

**Fig 4:**
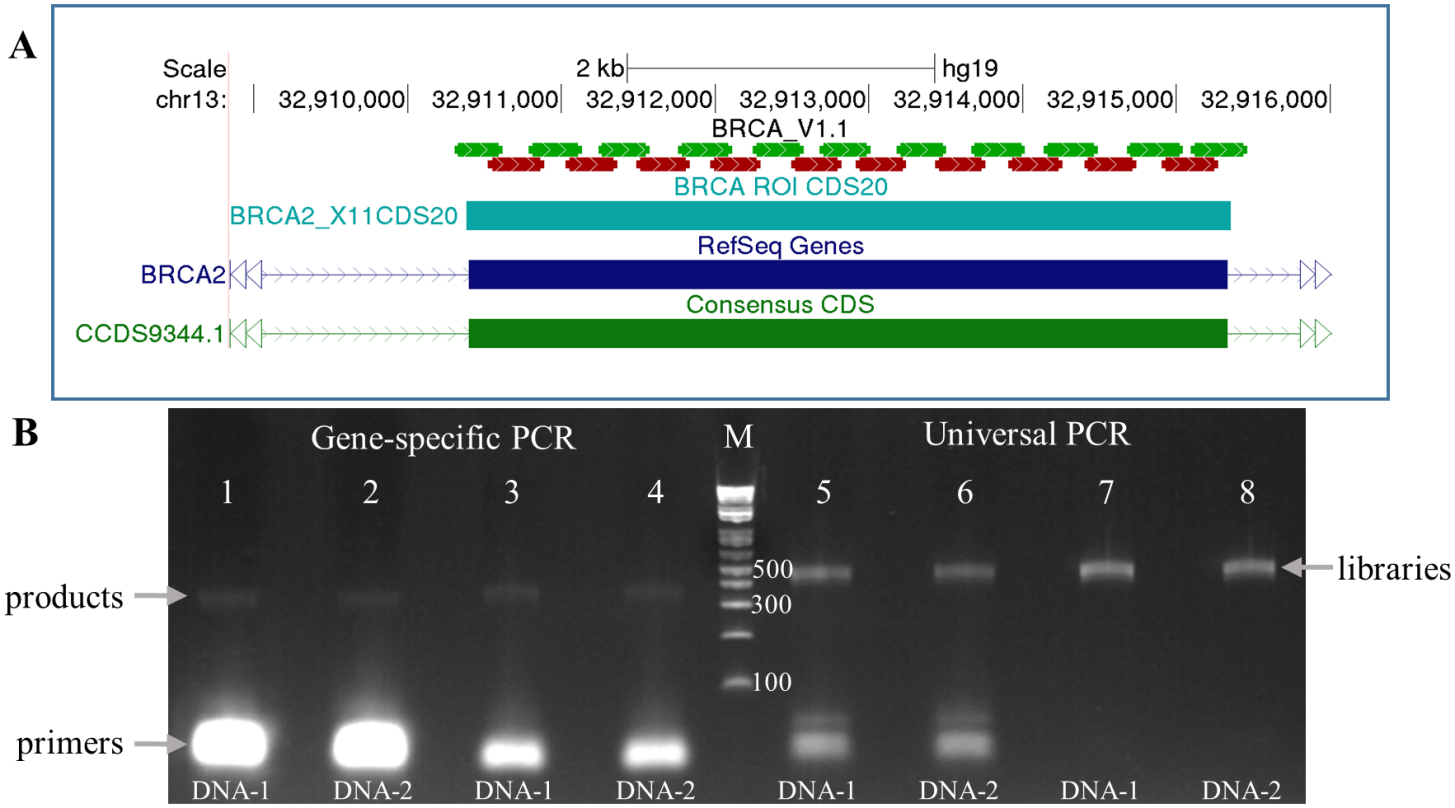
SLIMamp BRCA1 and BRCA2 library preparation. A) Example of hg19 positions of 21 overlapping amplicons for *BRCA2* exon 11. Oligonucleotides were designed to cover the entire coding regions of *BRCA1* and *BRCA2* ± 20 bp. The primer regions of each amplicon are represented with thin blocks. All primer binding regions in the exon are covered by their adjacent, overlapping amplicons to ensure 100% coverage of the entire targeted region (~5 Kb in size). The number of amplicons covering each coding exon for BRCA1 and BRCA2 is located in S1 Table. B) Gel electrophoresis of PCR products of two different samples at different library preparation steps. The gene-specific PCR products (lanes 1–4) with the SLIMamp BRCA oligos have an average size of 372 bp (average size of gene-specific region is 331 bp and additional 41 bp for TSCA tags). Bands that represent overlapping amplicons (90–234 bp with tags) are not seen on the gel either before (lanes 1 and 2) or after (lanes 3 and 4) bead purification. Libraries after universal PCR before (lanes 5 and 6) and after purification (lanes 7 and 8) have increased in size due to indexing, which is an additional 94 bp, resulting in an average size of 466 bp. Marker in Lane M is 1Kb Plus ladder.

Using the SLIMamp technology, oligonucleotides were designed to cover the coding regions (CDS ± 20 bp) of *BRCA1* and *BRCA2* (NM_007294.3 and NM_000059.3, respectively, see S1 Table). The ROI consists of 17,769 bp and is covered with 91 segments that range in size from 263–380 bp with an average of 331 bp. Out of the 91 amplicons, 53 amplicons overlap with at least one adjacent amplicon, resulting in a total of 45 overlapped regions that range from 49 bp to 193 bp in size. Fig 4A shows 21 overlapping amplicons that cover exon 11 (4.9Kb) of *BRCA2*.

Libraries for all 41 samples (additional sample information in S2 Table) were prepared using one pool of SLIMamp primers. An example of the initial gene-specific PCR products from two samples with 30 ng of input are shown in Fig 4B. The average insert size of the gene-specific regions is 331 bp, and with the additional tags for universal PCR, the average amplicon size is 372 bp, which is shown in lanes 1–4 in Fig 4B. Both before (lanes 1 and 2) and after (lanes 3 and 4) bead clean-up, PCR products did not contain overlapping amplicons, which would be present in the 90–234 bp range. The gene-specific PCR products underwent universal indexing PCR (lanes 5 and 6) for subsequent sample pooling and sequencing. The resulting libraries after a final purification are shown in lanes 7 and 8 (final average size of 466 bp). No overlapping amplicons were seen during the library preparation.

#### Mapping specificity and coverage uniformity

**Fig 5:**
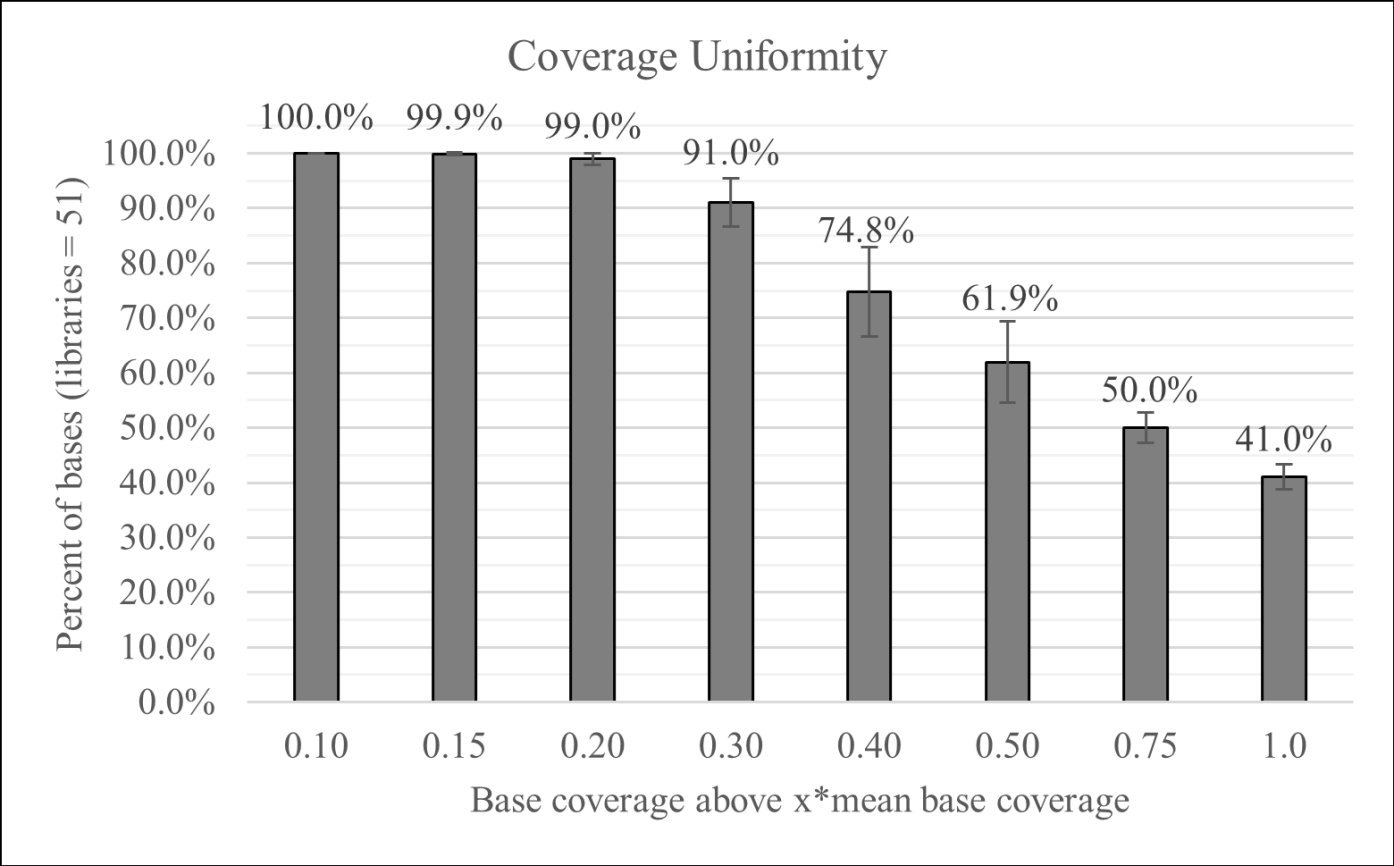
Coverage uniformity. 51 libraries were prepared from five independent runs. The SLIMamp BRCA assay produced consistent mapping, on-target rates, and uniformity (individual library statistics in S3 Table). The libraries had consistent coverage across amplicons without dropouts. (Mean ± St. Dev).

The 41 samples were used to prepare 51 libraries from five library preparations by three different operators. Due to the limited quantity of DNA from clinical samples, both 30 ng and 10 ng were used for DNA input to prepare the libraries. All samples were successfully amplified and their libraries sequenced successfully. The mean base coverage depth obtained from all the libraries ranged from 773x-8484x with a minimum base coverage (> = Q30) ranging from 152x-948x (S3 Table contains the individual library statistics). Overall, the libraries had a mapping rate of 99.62% (Coefficient of variance-CV: 0.59%) and an on-target rate of 99.85% (CV: 0.21%). The high mapping and on-target rates indicate the assay’s high specificity for the amplification of the target regions of *BRCA1* and *BRCA2*. During mPCR, amplification bias results in a range of coverage for different amplicons. The uniformity gives an indication of the coverage distribution (Fig 5) and is usually measured as 0.2x of the mean base coverage. The 51 libraries had 100% coverage at 0.1x relative to the mean coverage, indicating that all amplicons obtained coverage and none of the libraries had amplicon dropouts, and at 0.2x relative to the mean, the uniformity was 98.86% (CV: 0.61%). The sequencing data agreed with the gel results in which neither overlapping amplicons nor the long amplicons were represented in the reads (Fig 4B).

#### Variant detection concordance

The entire ROI of 34 unique samples, 32 Coriell samples and 2 clinical samples, were fully interrogated with independent methods (S2 Table). Among the samples, the mean base coverage ranged from 932-4166x with a minimum base coverage (> = Q30) from 182x-516x (individual coverage results in S3 Table). In the assay, 409 true positives were detected– 234 heterozygous SNVs, 153 homozygous SNVs, and 22 indels—and no false negatives, culminating in a sensitivity (TP/(TP+FN)) of 100% (95% CI: 99.10%-100.00%) (Table 3, full list of variants listed in S4 Table). In total, the 34 samples contain 66 unique variants: 4 insertions, 15 deletions, and 47 SNVs (Table 4). Additionally, no false positives were called, resulting in a specificity (TN/(FP+TN)) of 100% (95% CI: 100%-100%).

**Table 3:** Variant detection concordance.

Variant Detected				Variant not detected	
True Positive (TP)	409	SNV: HET	234	False Negative (FN)	0
		SNV: HOM	153		
		Indels: HET (1–40 bp)	22		
False Positives (FP)	0			True Negative (TN)*	603737

*TN = Size of ROI (17769 bp) x Total Samples (34)—Total True positives in all samples

**Table 4:** 66 Unique variants detected.

Type	Count	Notes
Insertion	4	1 bp
Deletion	15	1–4 bp, 11 bp, and 40 bp
SNV	47	
**Total**	**66**	

#### Reproducibility

To assesses the assay reproducibility, three of the samples, which included two Coriell DNA samples and one clinical sample, were run in three of the five library preparations with three different operators. One of the samples contains a 40 bp deletion, and the other samples contain two different 4 bp deletions. Among the three experiments, the nine libraries achieved a mapping rate of 99.24% (CV: 1.09%), an on-target rate of 99.84% (CV: 0.30%), and a coverage uniformity of 99.15% (CV: 0.76%) (S5 Table). The different variants in each sample were detected in each of the three runs with similar frequencies, and no discrepancies in variant detection were observed among the three runs (Table 5).

**Table 5:** Variant detection from independent library preparations.

					Run 1		Run 2		Run 3	
*Sample*	Gene	Exon/ Intron	c. Name	p. Name	Frequency (%) | Quality	Total Coverage | Normalized Coverage	Frequency (%) | Quality	Total Coverage | Normalized Coverage	Frequency (%) | Quality	Total Coverage | Normalized Coverage
*NA13707*	BRCA1	Exon10	c.1175_1214del40	p.Leu392GlnfsTer5	46.9 | 37	1459 | 0.43	49.7 | 37	2150 | 0.79	54.0 | 37	1589 | 0.63
			c.2082C>T		53.9 | 37	1156 | 0.32	48.0 | 37	2206 | 0.75	49.5 | 37	1163 | 0.43
			c.2311T>C		50.5 | 38	1015 | 0.28	45.4 | 38	1458 | 0.50	46.5 | 38	923 | 0.34
			c.2315T>C	p.Val772Ala	50.2 | 37	988 | 0.28	54.6 | 37	1419 | 0.50	53.8 | 38	900 | 0.34
			c.2612C>T	p.Pro871Leu	50.8 | 38	1256 | 0.35	50.3 | 38	1652 | 0.58	51.9 | 38	1160 | 0.44
			c.3113A>G	p.Glu1038Gly	50.2 | 38	835 | 0.23	49.3 | 38	1205 | 0.42	50.4 | 38	857 | 0.33
			c.3548A>G	p.Lys1183Arg	48.1 | 37	1537 | 0.42	47.3 | 37	1587 | 0.55	50.2 | 37	1322 | 0.50
		Exon12	c.4308T>C		50.5 | 38	9011 | 2.47	50.1 | 38	2727 | 0.93	50.1 | 38	4408 | 1.64
		Exon15	c.4837A>G	p.Ser1613Gly	50.5 | 38	3946 | 1.09	48.7 | 38	2779 | 0.96	48.9 | 38	2738 | 1.05
	BRCA2	Exon10	c.1114A>C	p.Asn372His	50.0 | 38	1174 | 0.32	48.9 | 38	1457 | 0.49	47.4 | 38	1166 | 0.43
		Exon11	c.3396A>G		50.3 | 38	1032 | 0.28	52.5 | 38	1348 | 0.46	47.8 | 38	938 | 0.35
			c.4563A>G		100 | 37	1064 | 0.29	99.9 | 37	1552 | 0.52	99.9 | 37	935 | 0.35
			c.6513G>C		98.0 | 33	1327 | 0.36	100 | 37	1407 | 0.47	99.8 | 36	993 | 0.36
		Exon14	c.7397T>C	p.Val2466Ala	99.8 | 35	3086 | 0.83	99.7 | 34	2456 | 0.83	99.7 | 35	2425 | 0.89
*NA14634*	BRCA1	Exon10	c.4065_4068delTCAA	p.Asn1355LysfsTer10	49.8 | 34	1531 | 0.38	49.1 | 34	1903 | 0.70	48.9 | 36	2093 | 0.60
	BRCA2	Exon11	c.3396A>G		51.0 | 38	1201 | 0.30	51.0 | 38	1306 | 0.48	51.8 | 38	1497 | 0.44
			c.4563A>G		99.9 | 37	1302 | 0.33	100 | 37	1540 | 0.57	100 | 37	1645 | 0.48
			c.5744C>T	p.Thr1915Met	52.3 | 36	968 | 0.28	48.1 | 36	950 | 0.42	52.6 | 37	1177 | 0.38
			c.6513G>C		98.2 | 33	1404 | 0.35	100 | 37	1332 | 0.48	99.9 | 36	1413 | 0.41
		Exon14	c.7242A>G		49.0 | 37	3248 | 0.82	48.0 | 37	2279 | 0.85	48.5 | 37	2940 | 0.86
			c.7397T>C	p.Val2466Ala	99.6 | 35	3292 | 0.82	99.3 | 33	2293 | 0.85	99.7 | 36	2969 | 0.86
		Intron 16	c.7806-14T>C		99.9 | 38	4932 | 1.29	99.9 | 38	4076 | 1.61	99.8 | 38	5049 | 1.54
*Clin-1*	BRCA2	Exon9	c.755_758delACAG	p.Asp252ValfsTer24	51.6 | 34	8396 | 2.04	52.6 | 35	1296 | 1.12	49.3 | 35	4685 | 1.59
		Exon10	c.1114A>C	p.Asn372His	99.8 | 38	1211 | 0.30	100 | 38	565 | 0.50	99.9 | 38	1385 | 0.47
		Exon11	c.4563A>G		99.9 | 37	1736 | 0.42	100 | 37	667 | 0.59	99.9 | 37	1397 | 0.47
			c.6513G>C		97.4 | 32	1429 | 0.34	100 | 37	540 | 0.46	99.9 | 35	1346 | 0.45
		Exon14	c.7397T>C	p.Val2466Ala	99.6 | 35	3186 | 0.78	99.9 | 34	887 | 0.77	99.8 | 36	2532 | 0.86
		Intron 16	c.7806-14T>C		48.1 | 38	4745 | 1.20	50.2 | 38	1622 | 1.45	49.8 | 38	4024 | 1.40

Three samples with previously confirmed variants had libraries prepared independently three times. The variants in the three samples were detected in all runs (NGS sample statistics in S5 Table).

#### DNA input range

To test the acceptable amount of DNA input, we used an input of 5–100 ng of DNA input (5, 10, 20, 30, 50, 75, and 100 ng) from four of the validated Coriell samples, including one DNA with a 40-bp deletion and another with an 11-bp deletion, to prepare 28 libraries. The libraries were prepared, pooled, and sequenced using an Illumina MiSeq with v2 chemistry. The libraries obtained a mean base coverage from 4582x-7435x and a minimum base coverage of 463x-1037x. The mean mapping rate was 99.04% (CV: 0.90%), and the on-target rate was 99.83% (CV: 0.07%) (Table 6). The libraries obtained a coverage uniformity of 98.51% (CV: 0.71%). The variants for each sample were 100% concordant for all input amounts. The input amount correlated with the final library yield, but it did not affect the NGS performance. For low input amounts (5–10 ng), the library yield can be increased by adding two additional cycles (5 vs 7 cycles) in indexing PCR without affecting the data quality or variant detection. This was verified in the reproducibility study described above in Run 2 with 10 ng input using 7 cycles of indexing PCR (Table 6 and S6 Table). Therefore, the SLIMamp BRCA assay allows for a range of input that can be tailored to sample availability.

**Table 6:** NGS statistics from 28 input libraries.

Parameter		Mean	Median	CV*	Minimum	Maximum
Mapping Rate		99.04%	99.40%	0.90%	96.44%	99.71%
On-Target Rate		99.83%	99.85%	0.07%	99.58%	99.90%
Coverage Uniformity		98.51%	98.66%	0.71%	96.97%	99.25%
Base coverage	Mean	6445	6489		4582	7435
	Median	4287	4272		3176	5578
	Maximum	26164	25752		16439	37138
	Minimum	831	838		463	1037
Percent of bases with coverage depth greater than Nx relative to mean base coverage	0.1x	100.00%	100.00%	0.00%	100.00%	100.00%
	0.15x	99.89%	100.00%	0.34%	98.90%	100.00%
	0.20x	99.49%	100.00%	0.69%	97.60%	100.00%
	0.30x	92.04%	92.50%	2.05%	87.60%	95.00%
	0.40x	74.82%	74.90%	3.30%	69.30%	79.40%
	0.50x	62.44%	62.70%	3.81%	56.50%	66.30%
	0.75x	47.86%	48.25%	4.30%	44.00%	50.90%
	1.0x	38.03%	38.45%	4.71%	33.00%	40.00%

Individual library statistics are shown in S6 Table.

*Coefficient of variance

#### Sample throughput

Sample pooling and sequencing depth require a balance between the number of samples that need to be processed and the amount of information needed for each sample. The ability to pool numerous samples for analysis reduces processing time while achieving sufficient depth is critical for analytical sensitivity. Based on the mapping rates and on-target rates observed in our study (S3 Table), 91,500 raw paired-end reads need to be obtained for each sample to achieve a mean amplicon coverage of 500x and a minimum amplicon coverage of 100x (0.2x of the mean coverage). Thus, a MiSeq Nano v2 kit, which produces about 2 million raw paired-end reads, could be used to run approximately 20 libraries, and fewer libraries will increase the minimum depth further (Table 7). MiSeq v2 and v3 kits produce 15 and 25 times more reads, respectively, than a MiSeq Nano v2 kit. Currently, Illumina’s TSCA Indexing kit allows for the dual-indexing of 96 libraries. Sequencing a pool of 96 libraries on a MiSeq v2 kit or MiSeq v3 kit would produce a mean amplicon coverage around 1700x and 2800x, respectively (Table 7). With custom indices to accommodate more than 96 libraries, sample throughput could be tailored for additional libraries on the MiSeq.

**Table 7:** Library multiplexing coverage.

MiSeq kit	Kit Raw paired-end reads*	Libraries	Projected minimum amplicon coverage^a^	Projected mean amplicon coverage^b^
Nano	2 million	20	100x	500x
v2	30 million	96	340x	1700x
v3	50 million	96	560x	2800x

Based on the data in Fig 5 and S3 Table, the sequencing data produced using the SLIMamp BRCA assay allows for a range of library pooling that can be tailored to various throughput needs based on the desired sequencing depth.

*Raw reads based on manufacturer’s reported reads for each kit

^a^Minimum amplicon coverage is taken to be 0.2x of the mean amplicon coverage.

^b^Coverage for each amplicon is taken from the amplicon non-overlapping region with merged reads (F/R reads on the same position count as one coverage).

## Discussion

We have developed SLIMamp PCR, a novel method for multiplex PCR, that enables amplification of overlapping amplicons in a single-tube format. SLIMamp inhibits amplification of overlapping amplicons by forming a stem that competes with primer annealing for extension. A portion of the forward primer sequence, (F2^) is added to the reverse primer, or vice versa (reverse sequence on the forward primer) that results in the overlapping amplicon forming a stem-loop structure. The stem reduces the availability of the sequence needed for primer annealing and with two-step cycling conditions during PCR, the amplification of the overlapping amplicon is inhibited.

SLIMamp PCR was used to amplify both *BRCA1* and *BRCA2* simultaneously in one multiplex reaction. The SLIMamp BRCA assay allows for single-tube NGS library preparations within six hours from extracted DNA, which significantly simplifies the library preparation process by reducing both the number of reactions and the amounts of reagents. Commercial kits such as the Ion AmpliSeq BRCA1 and BRCA2 Panel, Qiagen GeneRead Human BRCA1 and BRCA2 Panel, and Multiplicom BRCA MASTR Dx separate primer pairs into 3, 4, and 5 primers pools, respectively, due to the inability to amplify overlapping amplicons in multiplex PCR. Multiple primer pools significantly complicate the workflow, making them more error prone. RainDance Technologies avoids this issue by separating PCR primers into thousands of micro-droplets, but it requires a special, expensive instrument for micro-droplet PCR and amplicon pooling [10]. TruSeq Amplicon requires only one reaction pool per sample, but it utilizes hybrid capture followed by PCR [7].

Overall, this report provides a practical and economical single-tube multiplex PCR for targeted next generation sequencing. The SLIMamp BRCA assay did not have amplicon dropouts, evidenced with uniformity values >98%, and it consistently produced mapping and on-target rates >99%. Given this performance, the assay can be used to sequence multiple samples for tailoring to specific throughput needs. The assay also allowed for a range of input amounts without sacrificing data quality, and library preparations were consistent among different runs with regards to data quality and variant calls.

## Supporting information

S1 Table ROI. To cover the coding regions of BRCA1 and BRCA2 ± 20bp (17,769bp), 91 amplicons were designed. The hg19 position of each coding exon and the number of amplicons covering each coding region are shown; the largest exons (~3.4Kb and 4.9Kb) are covered with 15 and 21 amplicons, respectively.
(XLSX)

S2 Table Sample information. 41 samples were used to evaluate the SLIMamp assay performance. 33 of the samples were previously interrogated using Sanger sequencing. The 33 samples and Genome in a Bottle were used for the variant concordance study.
(XLSX)

S3 Table NGS statistics of 51 libraries in assay performance study. The individual mapping rates, on-target rates, and uniformity for each library indicated that coverage is consistent among the libraries, which were prepared by three operators in five different runs.
(XLSX)

S4 Table Unique variants detected in variant concordance study. Sixty-six unique variants were detected in 34 unique, samples by the SLIMamp BRCA assay.
(XLSX)

S5 Table NGS statistics of three samples in three independent runs. Nine libraries of three samples prepared three times with three operators. Both 10 ng and 30 ng DNA input were used, and the assay performance was similar among the three runs.
(XLSX)

S6 Table NGS statistics of DNA input libraries. Four Coriell DNA samples were used to prepared 28 libraries with seven input amounts ranging from 5ng-100ng.
(XLSX)
